# Comparison of the Trueness of Complete Dentures Fabricated Using Liquid Crystal Display 3D Printing According to Build Angle and Natural Light Exposure

**DOI:** 10.3390/jfb16080277

**Published:** 2025-07-30

**Authors:** Haeri Kim, KeunBaDa Son, So-Yeun Kim, Kyu-Bok Lee

**Affiliations:** 1 Department of Dental Science, Graduate School, Kyungpook National University, Daegu 41940, Republic of Korea; harry@dip.or.kr; 2 Advanced Dental Device Development Institute (A3DI), Kyungpook National University, Daegu 41940, Republic of Korea; oceanson@knu.ac.kr; 3 Department of Prosthodontics, School of Dentistry, Kyungpook National University, Daegu 41940, Republic of Korea

**Keywords:** complete denture, intaglio surface, trueness, LCD 3D printing, build angle, photopolymer resin, additive manufacturing, dimensional accuracy

## Abstract

The dimensional accuracy of the intaglio surface of complete dentures fabricated using liquid crystal display (LCD) three-dimensional (3D) printing might be influenced by the build angle and post-processing storage conditions. This study evaluated the effect of build angle and natural light exposure duration on the intaglio surface trueness of maxillary complete denture bases. Standardized denture base designs (2 mm uniform thickness) were fabricated using an LCD 3D printer (Lilivis Print; Huvitz, Seoul, Republic of Korea) at build angles of 0°, 45°, and 90° (*n* = 7 per group). All specimens were printed using the same photopolymer resin (Tera Harz Denture; Graphy, Seoul, Republic of Korea) and identical printing parameters, followed by ultrasonic cleaning and ultraviolet post-curing. Specimens were stored under controlled light-emitting diode lighting and exposed to natural light (400–800 lux) for 0, 14, or 30 days. The intaglio surfaces were scanned and superimposed on the original design data, following the International Organization for Standardization 12836. Quantitative assessment included root mean square deviation, mean deviation, and tolerance percentage. Statistical analyses were performed using one-way analysis of variance and paired *t*-tests (α = 0.05). Build angle and light exposure duration significantly affected surface trueness (*p* < 0.05). The 90° build angle group exhibited the highest accuracy and dimensional stability, while the 0° group showed the greatest deviations (*p* < 0.05). These findings underscore the importance of optimizing build orientation and storage conditions in denture 3D printing.

## 1. Introduction

The integration of digital technologies into dentistry, particularly computer-aided design (CAD) and computer-aided manufacturing, has significantly advanced the fabrication of dental prostheses, including complete dentures [1,2,3]. For edentulous patients, digital workflows offer enhanced fabrication accuracy, clinical efficiency, cost-effectiveness, reproducibility, and patient satisfaction compared to conventional analog techniques [4,5,6].

Digital complete denture fabrication is categorized into subtractive manufacturing and additive manufacturing (AM). AM has garnered attention due to its ability to minimize material waste, replicate complex anatomical geometries, and streamline production [7,8,9]. Common light-curing AM techniques include stereolithography (SLA), digital light processing (DLP), and liquid crystal display (LCD)-based three-dimensional (3D) printing [10,11,12]. Of these, LCD 3D printing has recently gained popularity due to its high resolution, rapid printing speed, and cost-effective scalability [13,14,15]. This technology uses a light-emitting diode (LED) light source projected through a liquid crystal panel to cure entire resin layers simultaneously, achieving precise layer formation with minimal optical distortion [16,17]. Its high resolution allows for accurate reproduction of complex geometries, making it particularly suitable for fabricating the intaglio surface of complete dentures, where precise replication of the edentulous oral mucosa is essential [18].

The accuracy of AM is influenced by multiple parameters, including build angle, layer thickness, exposure time, light intensity, and resin properties [19,20,21]. Among these, the build angle critically affects geometric fidelity by influencing the stair-stepping effect, stress distribution, and surface texture [22,23,24]. Accurate reproduction of the intaglio surface is particularly sensitive to build orientation due to its continuous curvature and functional role in mucosal contact. The intaglio surface is essential to the clinical performance of complete dentures, contributing to their retention, stability, and support [25,26]. According to the International Organization for Standardization (ISO) 5725-1, dimensional accuracy comprises two components: trueness, defined as the closeness of agreement between the average measurement and a reference value, and precision, defined as the variability among repeated measurements [27]. Of these, trueness is considered a key indicator of denture fit and long-term functionality [28].

While several studies have evaluated the trueness of denture bases fabricated using SLA and DLP technologies [29,30,31], limited data exist for LCD-based printing despite its increasing clinical use [11,32]. Prior research has shown that variations in build angle affect polymerization behavior and dimensional accuracy in DLP- [33] and SLA-based [34] AM systems. The potential influence of post-processing storage conditions, particularly natural light exposure, on dimensional stability has received limited attention. Residual photoinitiators and unreacted monomers in LCD resins might undergo continued polymerization under ambient light, potentially causing stress relaxation and deformation over time, thereby affecting the intaglio fit [35,36,37,38,39,40].

This study aimed to evaluate the effects of build angle (0°, 45°, and 90°) and natural light exposure duration (0, 14, and 30 days) on the intaglio surface trueness of maxillary complete dentures fabricated using LCD 3D printing. Using quantitative 3D deviation analysis, this study sought to provide evidence-based guidelines for optimizing fabrication and storage protocols to ensure clinical accuracy and dimensional stability. Two null hypotheses were formulated: (1) the build angle has no significant effect on intaglio surface trueness; (2) natural light exposure duration does not significantly affect intaglio surface trueness.

## 2. Materials and Methods

**Figure 1:**
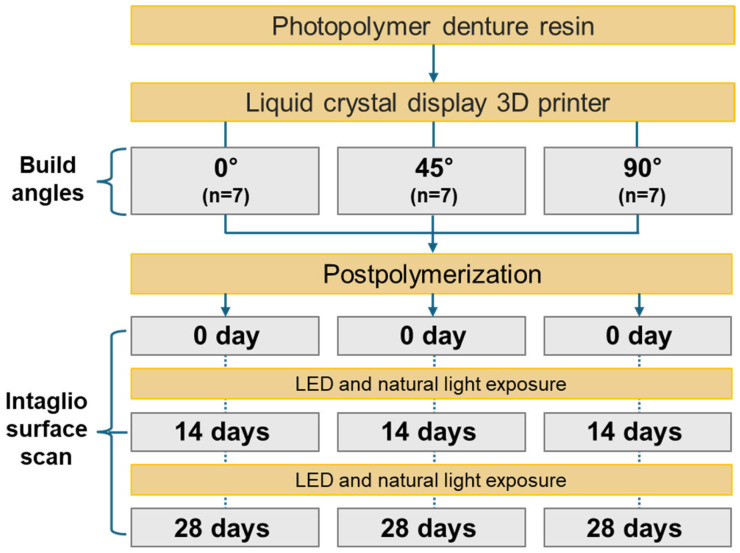
Overview of the experimental workflow to evaluate the impact of build angle and natural light exposure on the intaglio surface trueness of maxillary complete dentures fabricated using liquid crystal display 3D printing. Abbreviations: 3D, three-dimensional; LED, light-emitting diode.

**Figure 2:**
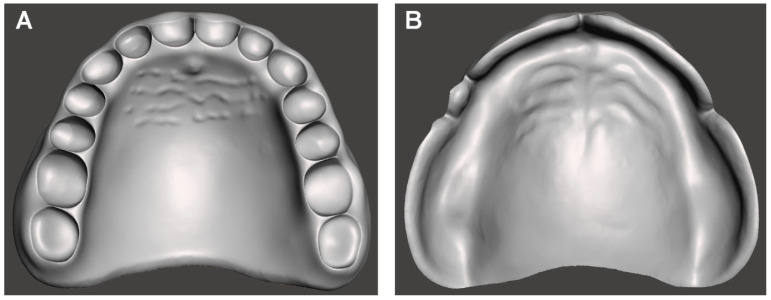
Computer-aided design model of the complete denture base used as the reference for trueness evaluation. (**A**) Polished surface view. (**B**) Intaglio (tissue-contacting) surface view.

This laboratory-based quantitative study aimed to evaluate the influence of build angle and natural light exposure on the intaglio surface trueness of complete dentures fabricated using LCD 3D printing (Figure 1). A commercially available edentulous maxillary resin model representing standard clinical anatomical morphology (Nissin Dental Products Inc., Kyoto, Japan) served as the reference. The model was scanned using a high-precision desktop scanner (E1; 3Shape A/S, Copenhagen, Denmark) at a resolution of 10 μm. The obtained data were imported into CAD software (Dental System 2020; 3Shape A/S) to design a maxillary complete denture base with a uniform thickness of 2 mm (Figure 2). The final design file was exported in standard tessellation language (STL) format and designated as the CAD reference model (CRM).

The STL file was processed using slicing software (Chitubox ver 2.3.1; Shenzhen, China) and categorized into three groups based on build angles: 0°, 45°, and 90°. In the 0° group, the polished surface of the denture base was aligned parallel to the build platform; the design was rotated counterclockwise accordingly for the 45° and 90° groups. All specimens were printed in a fixed orientation using identical parameters (Table 1). Support structures were automatically generated on the polished surface only, ensuring the intaglio surface remained undisturbed. Specimens were fabricated using an LCD 3D printer (Lilivis Print; Huvitz, Seoul, Republic of Korea) and a photopolymerizable denture base resin (Tera Harz Denture; Graphy, Seoul, Republic of Korea). Printing parameters were standardized across groups: 405 nm light source wavelength, 100 μm layer thickness, and 50 μm XY pixel size. Each group comprised seven specimens (*n* = 7). The sample size was determined through a priori power analysis using G*Power software (ver 3.1.9.7; Heinrich Heine University, Düsseldorf, Germany), based on an effect size (Cohen’s d) of 0.4, a statistical power of 0.8, and a significance level of α = 0.05. The effect size was derived from pilot data (*n* = 3 per group), which showed a mean root mean square (RMS) deviation difference of approximately 0.035 mm between the 90° and 0° groups, with a pooled standard deviation of approximately 0.085 mm, corresponding to an estimated Cohen’s d of 0.41. As previous studies lacked directly comparable quantitative data for LCD-printed complete dentures, the pilot data were used to inform the sample size estimation. A conservative medium effect size of 0.4 was selected to ensure adequate statistical power while minimizing overestimation risk.

Following fabrication, specimens were cleaned in 100% isopropyl alcohol using an ultrasonic cleaner for 3 min, then air-dried at room temperature for 10 min. Post-curing was performed for 5 min using an ultraviolet (UV) LED curing unit (Lilivis Cure; Huvitz) with an output power of 280 W and a peak wavelength of 395 nm. After polymerization, specimens were allowed to cool for 10 min and subsequently stored under ambient conditions (~23 °C, ~50% relative humidity). All post-processing procedures, including cleansing and post-polymerization, were performed according to the manufacturer’s instructions.

**Figure 3:**
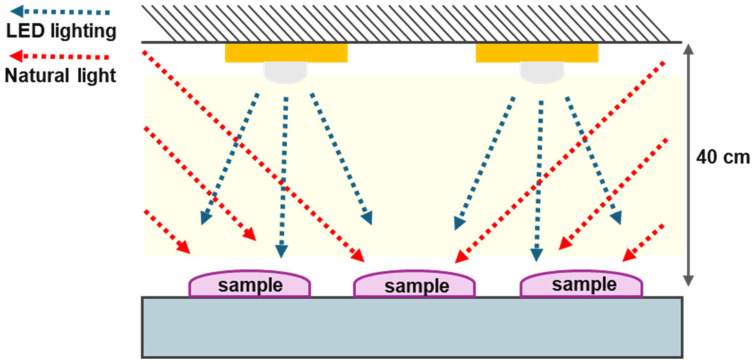
Schematic of the custom-designed lighting chamber used to simulate natural light exposure conditions. Abbreviations: LED, light-emitting diode.

All specimens were evaluated at three time points to simulate clinical storage conditions: immediately after scanning (day 0) and after storage for 14 and 30 days. Specimens were placed in an open-type lighting chamber equipped with ceiling-mounted LED lights (color temperature: 6500 K; wavelength range: 400–420 nm), with an average illuminance maintained at 3000 ± 150 lux, measured at the center of each specimen (Figure 3). The chamber also received ambient natural light through windows, providing additional exposure in the range of 400–800 lux (Figure 3). Illuminance was measured thrice daily using a digital lux meter (HS1010A; Benetech, Shenzhen, China), which follows the International Commission on Illumination photopic response curve and has a spectral sensitivity range of approximately 400–700 nm. Specimens were consistently positioned and oriented throughout the experimental period.

Before scanning, a scan spray (Ecoscan; High Dental, Seoul, Republic of Korea) was applied from a distance of 10–15 cm for 3 s to minimize surface reflection. Scanning was performed by a single trained operator using the same desktop scanner (E1; 3Shape A/S) under identical conditions. The resulting STL files were carefully examined for scanning errors. Only validated datasets were designated as CAD test models (CTMs). Before deviation analysis, STL files were preprocessed using mesh editing software (Geomagic Wrap; 3D Systems, Cary, NC, USA) for surface smoothing, abnormal vertex elimination, defect repair, and mesh standardization to approximately one million triangles.

**Figure 4:**
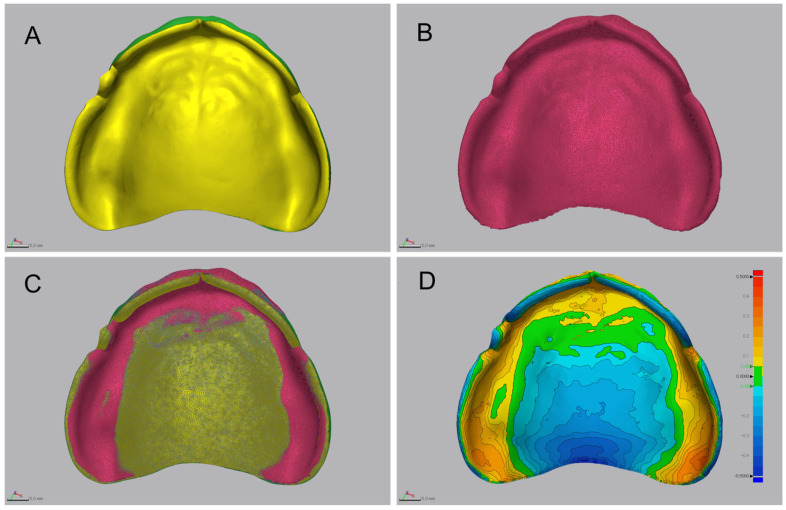
Workflow of the three-dimensional deviation analysis for evaluating intaglio surface trueness. (**A**) Computer-aided design (CAD) reference model (CRM) of the complete denture base. (**B**) Scanned CAD test model (CTM) after post-processing and surface preparation. (**C**) Alignment procedures comprising initial positioning and global best-fit alignment of the intaglio surface. (**D**) Color-coded deviation map illustrating surface discrepancies between CRM and CTM. The analysis was performed within a ±500 µm tolerance range; green areas indicate deviations within ±50 µm, representing clinically acceptable accuracy.

Three-dimensional deviation analysis between the CRM and each CTM was conducted using 3D inspection software (Geomagic Control X, version 2018.0.0; 3D Systems) following ISO 12836:2015 [41] (Figure 4). Initial alignment was based on a central palatal reference plane, followed by global best-fit alignment of the entire intaglio surface. Trueness was assessed using *RMS* deviation, mean positive deviation, mean negative deviation, and the percentage of points within a defined tolerance threshold (±50 μm). Surface deviations were visualized using a 20-step color map with a comparison range of ±500 μm, with deviations within ±50 μm displayed in green. The *RMS* deviation was calculated using the following Equation (1):

(1) $$RMS=\frac{1}{\sqrt{n}}\cdot \sqrt{\sum_{i=1}^{n}{\left(X_{1,i}-X_{2,i}\right)}^{2}}$$

where $X_{1,i\;}$ represents the *i*-th measurement point on the intaglio surface of the CRM, and $X_{2,i}$ corresponds to the measurement point on the CTM. The *RMS* value quantifies the mean absolute deviation between the two surfaces, providing an objective measure of trueness.

Statistical analysis was conducted using SPSS Statistics (version 29.0; IBM Corp., Armonk, NY, USA), with the significance level set at α = 0.05. The Shapiro–Wilk and Levene’s tests were used to verify the assumptions of normality and homogeneity of variances, respectively. As all parametric assumptions were satisfied, a one-way analysis of variance was used to examine differences among the build angle groups. When statistically significant differences were identified, post hoc comparisons were performed using Tukey’s honestly significant difference test. Paired *t*-tests were used for within-group comparisons across day 0, day 14, and day 30. The outcome variables included *RMS* deviation, mean positive deviation, mean negative deviation, and tolerance percentage. All results were interpreted for statistical significance and overall trends.

## 3. Results

### 3.1. Effect of Build Angle on the Intaglio Surface Trueness of Complete Dentures

**Figure 5:**
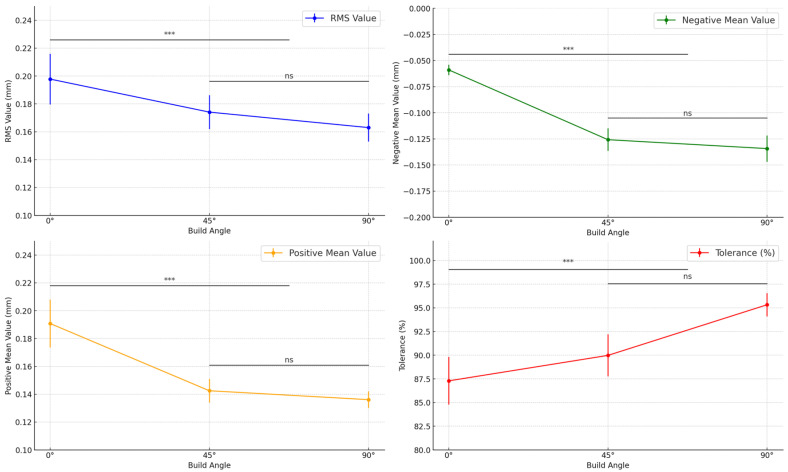
Comparison of intaglio surface trueness of complete dentures fabricated at different three-dimensional printing build angles. *** Indicates statistically significant differences (*p* < 0.05). Abbreviations: RMS, root mean square; ns, not significant.

The *RMS* values, representing the overall deviation between the fabricated intaglio surface and the reference model, differed significantly across the build angle groups (Table 2; Figure 5; *p* = 0.001). The 0° group exhibited the highest *RMS* value (0.1978 ± 0.01819 mm), whereas the 90° group showed the lowest (0.1630 ± 0.01003 mm), indicating that steeper build angles contributed to improved surface trueness.

Significant variation was also observed in the positive mean deviation, which quantifies localized protrusive errors on the intaglio surface (Table 2; Figure 5; *p* < 0.001). The 0° group exhibited the highest positive mean (0.1908 ± 0.01721 mm), whereas the 90° group had the lowest (0.1361 ± 0.00600 mm), suggesting that steeper build angles reduced excess material accumulation on convex regions.

The negative mean deviation, indicating inward contraction or under-contouring, also varied significantly across build angles (Table 2; Figure 5; *p* < 0.001). The 0° group had the smallest negative mean deviation (−0.0590 ± 0.00501 mm), whereas the 90° group exhibited the greatest contraction (−0.1343 ± 0.01257 mm), indicating that increased angles might intensify polymerization shrinkage in specific areas of the denture base.

The tolerance percentage, defined as the proportion of surface points within ±50 µm of the reference model, also showed statistically significant differences (Table 2; Figure 5; *p* < 0.001). The 90° group demonstrated the highest tolerance (95.32 ± 1.23%), whereas the 0° group showed the lowest (87.29 ± 2.52%), suggesting that steeper build orientations yield better dimensional accuracy within clinically acceptable thresholds.

### 3.2. Effect of Natural Light Exposure Duration on Intaglio Surface Trueness

**Figure 6:**
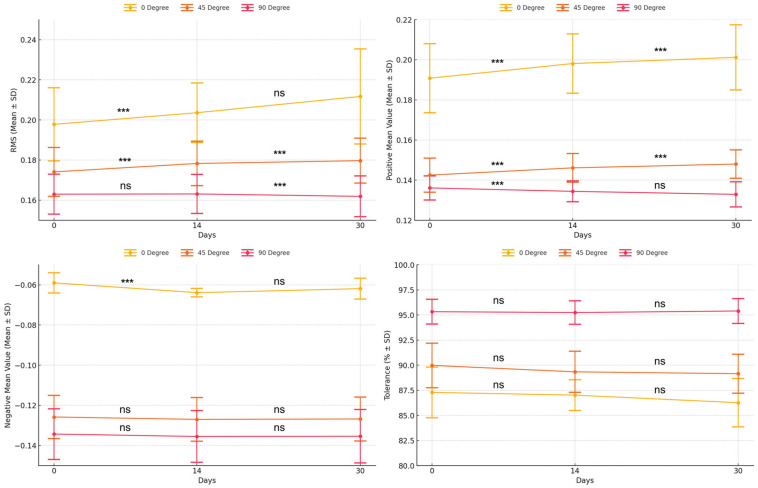
Comparison of intaglio surface trueness of complete dentures across different light exposure periods. *** Indicates statistically significant difference (*p* < 0.05). Abbreviations: RMS, root mean square; SD, standard deviation; ns, not significant.

Surface trueness, as measured by RMS values, deteriorated significantly following natural light exposure in specific build angle groups (Table 3; Figure 6). In the 0° group, the RMS values increased significantly from day 0 to day 14 (*p* = 0.011), with no further change observed by day 30 (*p* = 0.110). The 45° group exhibited progressive degradation, with significant increases between day 0 and 14 (*p* = 0.012) and between day 14 and day 30 (*p* = 0.007). In contrast, the 90° group showed a significant increase only between day 14 and day 30 (*p* = 0.044), with no change between day 0 and day 14 (*p* = 0.700).

The positive mean deviation increased significantly over time in all build angle groups (Table 3; Figure 6). For the 0° group, values increased between day 0 and day 14 (*p* = 0.002) and between day 14 and day 30 (*p* = 0.007). The 45° group showed similar patterns (*p* = 0.035 and *p* = 0.005, respectively). In the 90° group, a significant increase occurred only from day 0 to day 14 (*p* = 0.038), with no further change (*p* = 0.093).

In contrast, negative mean deviation showed minimal changes over time (Table 3; Figure 6). The 0° group exhibited a significant increase between day 0 and day 14 (*p* = 0.028) but no change thereafter (*p* = 0.340). Neither the 45° nor the 90° group demonstrated statistically significant differences over time (*p* > 0.05), suggesting that inward distortion remained relatively stable.

Tolerance values varied by the build angle group and exposure period (Table 3; Figure 6). The 0° group showed no significant differences between day 0 and day 14 (*p* = 0.556) or between day 14 and day 30 (*p* = 0.138). The 45° group demonstrated a significant decrease from day 0 to day 14 (*p* = 0.019), with no further reduction by day 30 (*p* = 0.076). In the 90° group, tolerance remained consistent throughout (*p* > 0.05), indicating preserved surface accuracy under extended light exposure in this orientation.

### 3.3. Qualitative 3D Deviation Map Analysis

**Figure 7:**
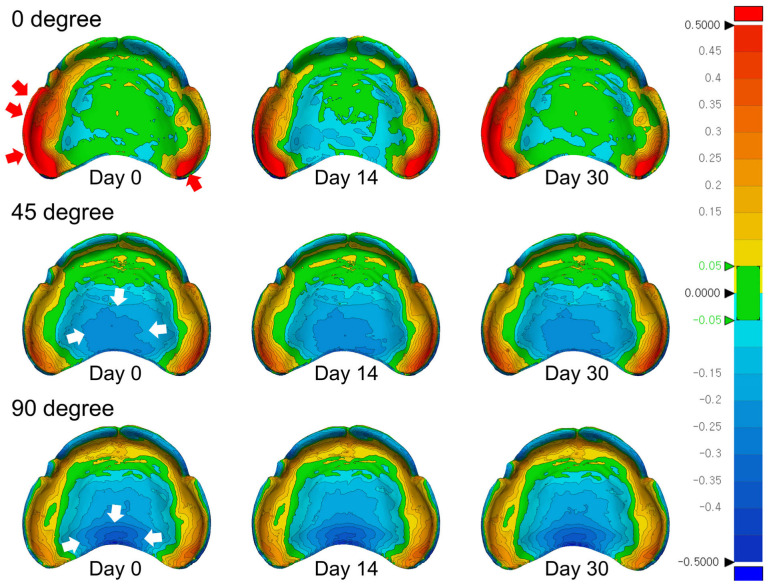
Three-dimensional color-coded deviation maps illustrating the intaglio surfaces of complete denture bases fabricated at different build angles (0°, 45°, and 90°) and evaluated at three time points (day 0, day 14, and day 30). Positive deviations (material protrusion relative to the reference model) are represented by red to yellow shades, while negative deviations (indicative of shrinkage or contraction) are depicted in green to blue. The color scale corresponds to a deviation range of ±0.5 mm.

Figure 7 illustrates color-mapped 3D deviation patterns of the intaglio surfaces across the three time points for each build angle group. In the 0° group, the initial assessment revealed pronounced positive deviations (red areas) along the buccal alveolar ridge and posterior palatal seal, indicating excessive material protrusion. By day 14, these areas showed reduced positive deviation, accompanied by the emergence of localized negative deviations (blue areas) in the mid-palatal zone. On day 30, the original protrusion pattern re-emerged in the buccal and posterior areas, suggesting cyclic dimensional behavior, possibly resulting from post-curing shrinkage, followed by stress relaxation or minor re-expansion.

In the 45° group, initial deviations were predominantly negative in the central palatal region. These areas exhibited progressive changes through day 14 and day 30, indicating continued surface distortion in response to environmental exposure within the palatal vault.

The 90° group consistently demonstrated negative deviations in the posterior palate and lingual alveolar ridge throughout the evaluation period. The persistence of contraction in these regions suggests that while the 90° orientation might offer superior initial trueness, certain anatomical zones remain susceptible to long-term dimensional changes under ambient light exposure.

## 4. Discussion

This study evaluated the effects of build angle and natural light exposure on the intaglio surface trueness of maxillary complete dentures fabricated using an LCD-based photopolymerization 3D printing system. The null hypothesis—that neither build angle nor natural light exposure would significantly influence intaglio surface trueness—was rejected, as both factors had statistically significant effects (*p* < 0.05) on dimensional accuracy. Among the tested conditions, the 90° build orientation demonstrated the lowest RMS deviation and the greatest dimensional stability, immediately after post-curing and following extended light exposure.

Build orientation plays a critical role in AM by affecting polymerization shrinkage, gravitational deformation, and the interaction with support structures. The 90° orientation aligns the stacking direction with the gravitational axis, thereby reducing unsupported overhangs and vertically distributing polymerization stresses. These results align with prior findings from SLA and DLP systems [11,14,35,36] and extend the evidence to LCD-based systems. Dentures fabricated at 90° consistently demonstrated intaglio surface trueness within the clinically acceptable threshold of ±100 µm [1,35], with over 95% of the surface area within ±50 µm. The ±50 µm threshold was adopted based on prior studies that have established this range as clinically acceptable for evaluating denture fit and adaptation. Previous studies, including those by Charoenphol et al. [42] and Tsai et al. [43], have used a ±50 µm threshold to evaluate the trueness of 3D-printed denture bases. This criterion not only reflects a level of dimensional discrepancy unlikely to affect clinical performance but also facilitates consistent and interpretable visualization in color-coded deviation maps. Prior studies [44,45] have reported that intaglio surfaces with 3D surface deviations of <100 µm generally exhibit excellent tissue adaptation, without compromising denture retention, stability, or support. Based on these findings, a mean deviation of approximately 30 µm is considered well within clinically acceptable limits, particularly in the palatal region, where soft tissue resilience can accommodate minor discrepancies.

In contrast, the 0° build angle involves horizontal layering across extensive surface areas, which promotes non-uniform polymerization shrinkage and the accumulation of stair-step artifacts, especially in anatomically flat regions, such as the posterior palatal area. Similar patterns of increased deviation have been reported in studies using other photopolymerization systems [12,18]. The 45° orientation, often recommended as a compromise between geometric accuracy and printability, demonstrated greater trueness variability in the present study. This might be attributed to asymmetric load distribution and localized distortion during post-curing, as noted by Shin et al. [40]. The dimensional degradation observed in the 45° group is likely due to anisotropic polymerization shrinkage and uneven stress distribution caused by the oblique stacking orientation. This configuration results in asymmetric support loading and localized distortion during post-curing, potentially intensified by thermal accumulation and the continued activation of residual photoinitiators under ambient light exposure. Specifically, the 45° build angle generates irregular cross-sectional exposure and non-uniform resin thickness, increasing the likelihood of localized light penetration and post-polymerization shrinkage due to uneven activation of residual photoinitiators. In contrast, the 90° orientation aligns the layers vertically, facilitating more uniform stress release and minimizing unsupported overhangs.

The 90° build orientation, characterized by vertically aligned layers, facilitates more uniform stress distribution and reduces the occurrence of unsupported overhangs. This structural configuration contributes to enhanced dimensional stability, as evidenced by consistent performance across immediate and delayed assessments in the present study.

Notably, post-curing exposure to natural light had a significant effect on the dimensional stability of printed dentures, particularly in the 0° and 45° groups. Prolonged exposure to ambient light may reactivate residual photoinitiators, inducing further polymerization and subsequent shrinkage [38,39]. These results align with prior findings by Kim and Lee [39] and Benfaida et al. [38], which highlight the risks associated with uncontrolled post-processing conditions. The evaluation time points of 14 and 30 days were selected to simulate clinically relevant durations of temporary denture use, as interim complete dentures are commonly worn for 2 to 4 weeks during post-extraction or post-implant healing periods [46]. This study demonstrated a significant increase in RMS deviation after 14 days of light exposure in the 0° and 45° groups, whereas the 90° group retained its dimensional accuracy. These findings underscore the importance of standardized post-curing and storage protocols, including the use of UV-blocking containers or inert gas environments, to prevent dimensional changes over time.

Clinically, the trueness of the denture intaglio surface is essential for achieving optimal adaptation to the mucosa, thereby improving prosthesis retention, patient comfort, and minimizing the need for chairside adjustments. Previous studies by Özden et al. [1] and Lee et al. [35] suggested that internal misfits exceeding 100 µm may compromise clinical performance. The superior accuracy observed in the 90° group suggests that vertical build orientation might offer a reliable approach for fabricating well-adapted complete dentures using LCD 3D printing. While geometric accuracy is paramount, esthetic considerations are also relevant. Temizci and Kölüş [4] reported that dentures printed at 90° demonstrated greater color discrepancies compared to other orientations, possibly due to variations in surface energy and light penetration during polymerization. Therefore, selecting an appropriate build angle requires balancing dimensional accuracy and the optical properties of the final restoration [4,20,35]. From a manufacturing standpoint, the 90° orientation is advantageous for support efficiency and reduced material consumption; however, it may require extended print durations due to the increased vertical build height [32]. Studies by Ko et al. [23] and Subbaiah et al. [30] emphasized the complex interaction between build angle, layer thickness, and exposure parameters, which collectively influence fabrication time and accuracy. Although this study maintained a consistent layer thickness across groups, future investigations should explore multi-factorial optimization strategies to enhance production efficiency and prosthesis quality.

Subtractive manufacturing techniques, such as milling, have traditionally been considered more accurate; however, recent advancements in AM have reduced this disparity. Studies by Lo Russo et al. [28] and Negm et al. [31] demonstrated that additively manufactured prostheses can achieve clinically acceptable adaptation when optimal build parameters are employed. The present findings further support the potential of LCD-based printing for complete denture fabrication, provided that critical parameters, particularly build orientation and storage conditions, are precisely controlled.

This study has certain limitations. First, all experiments were conducted under in vitro conditions, which do not fully simulate intraoral variables, such as moisture, temperature fluctuations, and functional loading. Second, the study used only a single commercially available denture base resin, limiting the generalizability of the results to other materials. Third, the analysis focused solely on intaglio trueness without assessing surface roughness, mechanical properties, or long-term aging behavior. Moreover, material-dependent behavior and patient-specific anatomical variability were not considered, which might affect clinical outcomes. Future studies should incorporate comprehensive performance metrics, a broader range of materials, patient-derived anatomical models, and in vivo assessments to enhance the clinical applicability of the findings.

In conclusion, this study demonstrated that build orientation and natural light exposure significantly affect the intaglio surface trueness of complete dentures fabricated using LCD-based 3D printing. The null hypothesis was rejected, as the 90° build orientation consistently exhibited superior trueness and dimensional stability compared to the 0° and 45° orientations, even after prolonged environmental exposure. These findings provide critical insights for optimizing AM protocols in prosthodontics and developing reliable digital workflows for complete denture fabrication. The findings suggest that careful selection of build angle and controlled post-processing light exposure can directly improve the fit accuracy of 3D-printed complete dentures. Incorporating these parameters into digital denture workflows might reduce chairside adjustment time and enhance overall patient satisfaction. Further clinical validation is warranted to establish standardized manufacturing guidelines and evaluate patient-centered outcomes, including retention, comfort, and functional performance.

## 5. Conclusions

Within the limitations of this study, the 90° build angle exhibited the highest intaglio surface trueness and dimensional stability, rendering it the most favorable orientation for 3D printing of complete dentures. The 45° build angle yielded clinically acceptable accuracy but exhibited greater susceptibility to dimensional degradation over time. Conversely, the 0° build angle produced the largest deviations, resulting in the poorest surface accuracy. Additionally, natural light exposure significantly affected surface accuracy, particularly during the early post-curing period. These findings suggest that optimizing build orientation and minimizing uncontrolled light exposure enhance the precision and clinical fit of 3D-printed complete dentures. Based on these results, a 90° build orientation is recommended to achieve optimal trueness and long-term dimensional stability in digital denture fabrication. However, further in vivo studies are warranted to validate the clinical applicability and generalizability of these findings.

## Figures and Tables

**Table 1:** Specifications of the 3D printing system, materials, and printing parameters.

Category	Item	Specification
3D printer	Device	Lilivis Print (Huvitz)
	Printing type	Adaptive MSLA
	Light source (printer)	405 nm
	Pixel size (X/Y)	50 µm
Curing unit	Light source	395 nm UV LED
	LED output power	280 W
Material	Material name	Tera Harz Denture (Graphy)
	Lot number	1-EB07D02048/1-EE01D02065
Scanner	Model	E1 (3Shape)
	Software	3Shape Dental System, version 17.3.0
Printing parameters	Layer thickness	100 µm
	Bottom layer count	5
	Transition layer count	10
	Bottom exposure time	10 s
	Bottom lift distance	5 mm
	Bottom lift speed	200 mm/min
	Bottom retract speed	150 mm/min
	Bottom light intensity	75%
	Normal exposure time	2 s
	Normal lift distance	5 mm
	Normal lift speed	80 mm/min
	Normal retract speed	150 mm/min
	Normal light intensity	40%
	Rest time before lift	0 s
	Rest time after lift	0 s

Abbreviations: 3D, three-dimensional; MSLA, masked stereolithography; UV, ultraviolet; LED, light-emitting diode.

**Table 2:** Comparison of intaglio surface trueness of complete dentures fabricated at different three-dimensional printing build angles.

Measurement Value	Build Angle	Mean	SD	95% Confidence Interval		Minimum	Maximum	F	*p*
				Lower	Upper				
RMS value (mm)	0°	0.197 ^A^	0.018	0.1809	0.2146	0.17	0.23	11.400	0.001 *
	45°	0.174 ^B^	0.012	0.1628	0.1853	0.15	0.19		
	90°	0.163 ^B^	0.010	0.1537	0.1723	0.15	0.18		
Positive mean value (mm)	0°	0.190 ^A^	0.017	0.1749	0.2067	0.17	0.22	46.507	<0.001 *
	45°	0.142 ^B^	0.008	0.1347	0.1503	0.13	0.16		
	90°	0.136 ^B^	0.006	0.1306	0.1417	0.13	0.14		
Negative mean value (mm)	0°	−0.059 ^A^	0.005	−0.0636	−0.0543	−0.06	−0.05	119.162	<0.001 *
	45°	−0.1258 ^B^	0.010	−0.1358	−0.1158	−0.14	−0.1		
	90°	−0.1343 ^B^	0.012	−0.1459	−0.1227	−0.16	−0.12		
Tolerance (%)	0°	87.291 ^A^	2.521	84.9593	89.6241	82.54	90.32	27.423	<0.001 *
	45°	89.978 ^A^	2.218	87.9263	92.0304	86.37	93.7		
	90°	95.326 ^B^	1.235	94.1832	96.469	92.89	96.76		

* Indicates statistical significance determined using one-way analysis of variance (*p* < 0.05). Uppercase letters indicate statistically significant differences among groups based on Tukey’s honestly significant difference test; groups sharing the same letter are not significantly different. Abbreviations: SD, standard deviation; RMS, root mean square.

**Table 3:** Comparison of intaglio surface trueness of complete dentures across different light exposure periods.

Build Angle	Comparison	Measurement Value	t	*p*
0°	Day 0 vs. day 14	RMS value	−3.591	0.011 *
		Positive mean value	−5.295	0.002 *
		Negative mean value	2.882	0.028 *
		Tolerance	0.624	0.556
	Day 14 vs. day 30	RMS value	−1.873	0.110
		Positive mean value	−4.062	0.007 *
		Negative mean value	−1.036	0.340
		Tolerance	1.713	0.138
45°	Day 0 vs. day 14	RMS value	−3.575	0.012 *
		Positive mean value	−2.705	0.035 *
		Negative mean value	2.092	0.081
		Tolerance	3.177	0.019 *
	Day 14 vs. day 30	RMS value	−3.981	0.007 *
		Positive mean value	−4.251	0.005 *
		Negative mean value	−0.327	0.755
		Tolerance	2.146	0.076
90°	Day 0 vs. day 14	RMS value	−0.404	0.700
		Positive mean value	2.653	0.038 *
		Negative mean value	2.176	0.073
		Tolerance	1.12	0.305
	Day 14 vs. day 30	RMS value	2.544	0.044 *
		Positive mean value	1.995	0.093
		Negative mean value	−0.391	0.709
		Tolerance	−2.167	0.073

* Indicates statistical significance determined by a paired *t*-test (*p* < 0.05). Abbreviation: RMS, root mean square.

## Data Availability

The original contributions presented in the study are included in thearticle, further inquiries can be directed to the corresponding author.
